# Compact high-efficiency energy harvesting positive and negative DC supplies voltage for battery-less CMOS receiver

**DOI:** 10.1038/s41598-023-41236-9

**Published:** 2023-08-30

**Authors:** Marwa Mansour, Islam Mansour

**Affiliations:** 1 Microelectronics Department, Electronics Research Institute (ERI), El Nozha, Cairo 11843 Egypt; 2 Electrical Engineering Dept, Shoubra Faculty of Engineering, Benha University, Cairo, 11629 Egypt

**Keywords:** Electrical and electronic engineering, Energy harvesting

## Abstract

In this paper, novel compact high-efficiency multi-band rectifiers that supply positive and negative output voltages are demonstrated for energy harvesting applications. The proposed voltage doubler circuits are used as real DC voltage supplies of radio frequency mm-wave CMOS receivers. Operating multi-band rectifiers have a complicated structure that required more resonance networks to force the rectifier to work in multi-band. Novel series and parallel resonance networks are implemented to force the rectifier to operate in dual-band at frequencies of 850 and 1400 MHz. The proposed resonance network eliminates the Schottky diode impedance variation as the input power or frequency changes and supports the impedance matching and minimizes the insertion loss. A novel high-quality sine-shape micro-strip inductor that obtains a quality factor above 65 over the frequency band from 200 to 1400 MHz and inductance equal to 14 ± 2 nH is designed to improve efficiency and enhance performance at low power levels. The first suggested RF voltage doubler rectifier with series resonance feedback between the input and cathode of the diode and parallel resonance operates at two frequency bands of 850 and 1400 MHz and obtains a peak conversion efficiency of 59%, a saturated output DC voltage is 2.5 V, and the conversion efficiency is 40% at RF-input-power of − 10 dBm. This voltage doubler achieves the required DC supply parameter (1.1 V and 450 uA) for biasing the mm-wave receiver at an RF input power of 0 dBm. Otherwise, the second suggested negative voltage rectifier has a maximum simulated conversion efficiency of 65%, saturated negative DC-voltage is − 3.5 V, and the conversion efficiency is 45% at an RF input power of − 10 dBm. The negative voltage rectifier obtains DC supply parameters (− 0.5 V and no current condition used for a gate bias) at − 10 dBm input power.

## Introduction

The most important topics in radio frequency research are energy harvesting (EH) and wireless power transfer (WPT). Telecommunication systems with high power levels and power transmission over large distances are more convenient to use WPT. Whereas systems with low power levels are best suited to use energy harvesting (EH). The use of batteries in low-power devices/systems is eliminated by providing ambient radio frequency energy harvesting, such as in the internet of things (IoT) technology. Due to the fast expansion of wireless techniques, electromagnetic energy sources such as WiFi, ISM devices, and cellular networks become more and more available and eligible for energy harvesting^1^. In order to gather the most energy possible, the EH rectifiers must operate in a wideband or multi-band. Nevertheless, it is challenging to design multi-band^2^ or broadband rectifiers^3^ with high conversion efficiency and a wide input power range. The reason comes from the nonlinearity variation of the diode impedance with the frequency and RF input power. So, complicated matching circuits are needed, which leads to extra insertion loss and poor RF–DC efficiency.

Moreover, there is a lot of research in designing radio frequency rectifiers for example a reconfigurable class-F voltage doubler and two-stage voltage doubler at 650 and 900 MHz, and this work’s authors concentrate on the DC output voltage only and the circuit is very complicated meanwhile does not mention anything about the current^4^. A wideband radio frequency rectifier depends on microstrip transmission lines (TL) structure that occupies a large PCB size of 40 × 25 mm^2^, the maximum efficiency and the DC output voltage are achieved at high RF input power of 15 dBm not appropriate for energy harvesting applications^5^ therefore making it inappropriate for ambient energy harvesting applications. Whereas a 0.87–2.5 GHz RF rectifier was presented in^6^, this achieved a low efficiency of 30% at 0 dBm input power, and the DC output voltage was not mentioned in the paper. Ref^5,6^ didn’t talk about the rectifier current, only interested in the DC output voltage and consuming a large area. In ref^7,8^ energy harvesting (EH) systems were presented to enable the long-term recharge-free operation of IoT devices and applications. In the ref^9^ a wideband voltage doubler rectifier using a π-section network and series LC circuit, increasing the complexity of the design was fabricated by the authors and obtained RF–DC conversion efficiency above 69% in the frequency band from 720 to 1050 MHz, and the input reflection coefficient ($${S}_{11})$$ is less than − 10 dB at an input power value of 3 dBm. While in ref^10^ a complicated T-section that consists of a parallel LC circuit in the two arms was utilized to operate the rectifier in dual-band. The modeling of the Schottky diode and the analysis of the impedance aware rectifier sizing are explained in^11,12^.

In ref^13^ a dual resonant rectifier using a coupled resonator with series and parallel resonators was implemented to obtain a double-band circuit and combine it with standard LC matching. This made the design very complex and occupies a very large PCB area and needs a large number of lumped components. This achieved an RF-DC efficiency of 17.3% and 7.5% at − 10 dBm. While the DC voltages are equal to 436 mV and 286 mV at 490 MHz and 860 MHz respectively for single-tone measurements. However, a low complex single diode co-planer rectifier was introduced that had low efficiency equal to 22.5% @ − 19 dBm. Meanwhile, it worked at a single frequency band of 868 MHz and there is no information about the DC output voltage and size^14^. In ref^15^ a compacted single-diode implantable rectenna was presented at 673 MHz for medical applications. The rectifier circuit obtained an RF–DC conversion efficiency of 40% at an input power equal to − 20 dBm. In ref^16^ a rectifier circuit for far-field wireless powering was suggested and achieved an efficiency of 44% at an input power of − 10 dBm. A dual-band single-diode rectifier for GSM1800 and UMTS Band 1 was fabricated and achieved an efficiency of about 45% for the UMTS Band 1 and 33% for the GSM1800 when the incident power is − 7 dBm^17^. There is very little work related to the negative voltage topology as^18^ a DC Polarity control in RF Rectifier networks that generated a positive and negative by tuning switching device (GaN class-F^−1^ rectifier) but operated at very high input power from 20 to 40 dBm results a DC output voltage from 0 to ± 20 V. In ref^19^ a half-wave rectifier with positive and negative voltage was built employing OTA ICs (LT1228) and had DC output voltage from 0 to ± 5 V. Ref^20^ active rectifiers with positive and negative DC output voltage from ± 400 to ± 200 V. The above published works produced negative DC voltage by different methods than the introduced in this article.

In this paper, the proposed voltage doubler circuits are used as real DC voltage supplies of radio frequency receivers, where the positive voltage doubler is the main DC supply and the negative voltage doubler is used as a gate bias of the amplifier stage (PMOS Transistor) in the RF receiver. Series and parallel resonance networks are implemented to force the rectifier to operate in dual-band at frequencies of 850 and 1400 MHz. A novel series resonance feedback circuit between the input and cathode of the Schottky diode was added to produce an additional operating frequency band. A novel high-quality factor Sin-Shape microstrip inductor that obtains a quality factor above 65 over the frequency band from 200 to 1400 MHz and inductance equal to 14 ± 2 nH is implemented and used in the proposed voltage doubler designs to improve the efficiency and enhance the performance at low power level.

## Proposed positive DC supply voltage

The proposed positive voltage doubler is used as a DC supply voltage of the radio frequency (RF) receiver, where the RF receiver needs two DC supply voltages with the following specification, 1.1 V with 450 uA and − 0.5 V with very low ampere current (used as a bias on the transistor gate).

### Proposed positive voltage doubler design

**Figure 1:**
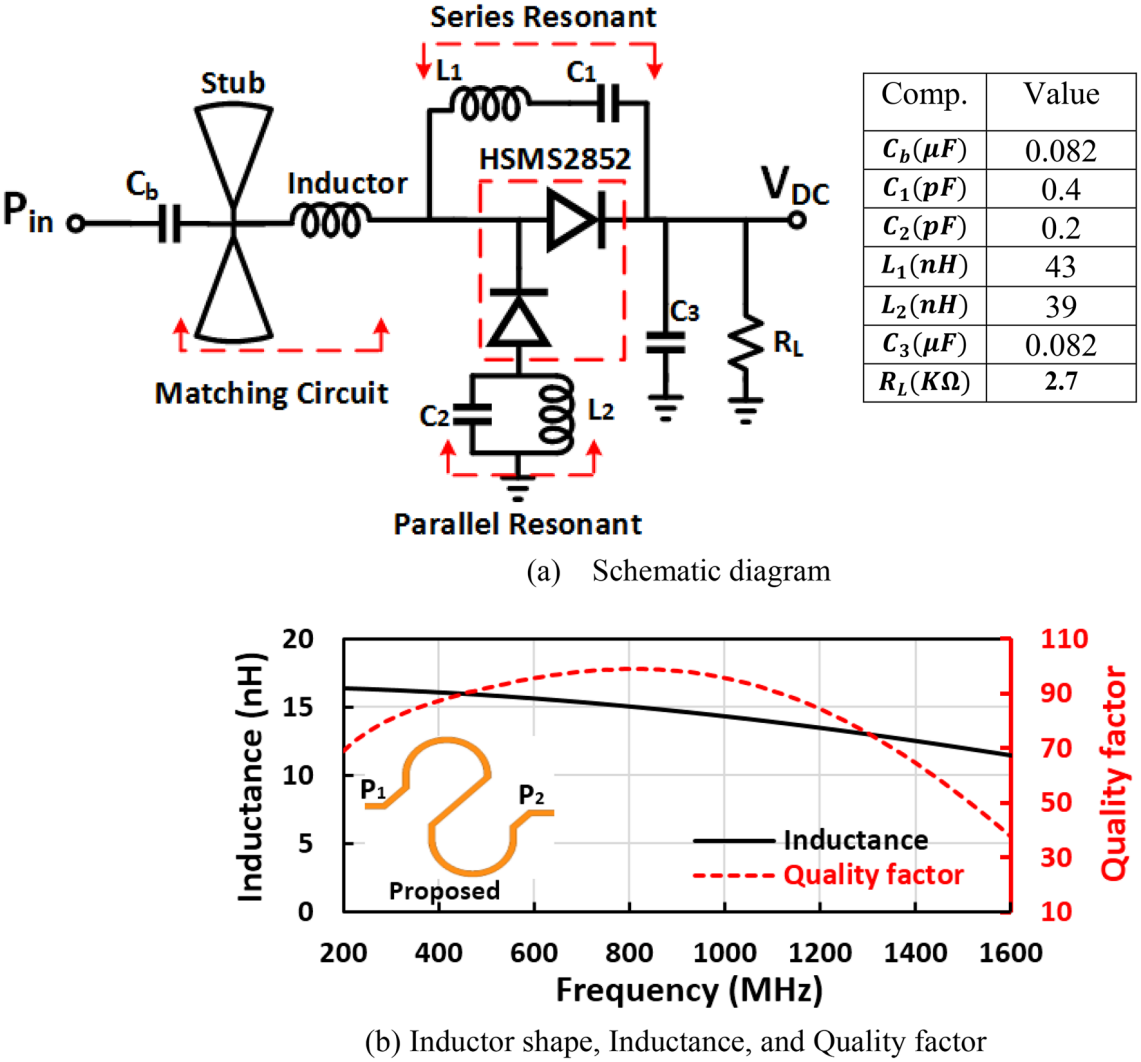
(**a**) Circuit schematic of the suggested RF positive voltage doubler and (**b**) electromagnetic simulation results of the suggested micro-strip inductor.

**Figure 2:**
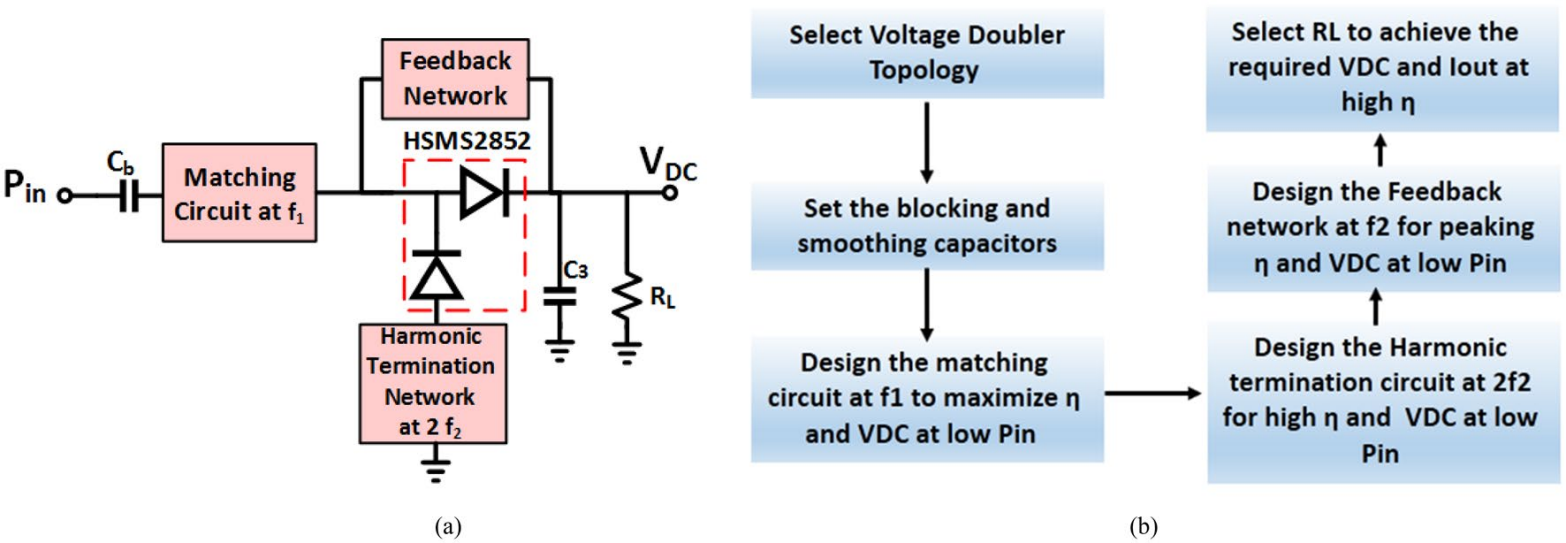
(**a**) Block diagram, and (**b**) step-by-step design sequence of proposed dual-band positive voltage doubler.

As displayed in Fig. 1a, the first suggested voltage doubler is composed of an input matching network, a series resonance feedback circuit, a parallel resonance network, voltage doubler diodes, capacitors for smoothing and charging, and load resistance. The Schottky diode in the suggested RF voltage doubler is chosen to utilize in low-power systems i.e. obtain maximum efficiency with agreeable DC output voltage at low input power to be appropriate for energy harvesting systems. Figure 2a illustrates the block diagram of the proposed positive dual band voltage doubler, where the figure illustrates the function of each sub-circuit/part in the proposed positive voltage doubler. Figure 2b Summarizes the design sequence for the positive dual band voltage doubler in six steps including the design architecture, set the blocking and smoothing capacitors values, design of the first band matching at ${f}_{1}$, design of the harmonic termination network and feedback network for matching the second band at ${f}_{2}$ and final step select the value of the load terminal resistance. Where *f*_1 _= 1400 MHz and *f*_2 _= 850 MHz in the proposed design.The parallel resonance circuit (${L}_{2},{C}_{2}$) was used to compensate for the capacitive input impedance of the diode anode by inserting an inductive reactance ${\mathrm{jZ}}_{01}$ at the fundamental frequency ${f}_{o}(0.85 \mathrm{GHz}).$ As well as creating an open circuit at the second harmonic frequency (${2f}_{o}$) to eliminate the second harmonic current component as illustrated in Eq. (1),

1 $$\begin{array}{*{20}c} {Z_{p} = \left\{ {\begin{array}{*{20}c} {0,} & {f = 0} \\ {jZ_{01} ,} & {f = f_{0} } \\ {\infty ,} & {f = 2f_{0} } \\ { - jZ_{01} ,} & {f = 3f_{0} } \\ \end{array} } \right.{ }} \\ \end{array}$$

where ${\mathrm{Z}}_{01}$ is the impedance of parallel resonance circuit at the fundamental frequency ${f}_{o}$.

For the second harmonic termination network, we selected the inductance value of ${L}_{2}$ and the capacitance value is specified by the resonance circuit condition.

2 $$C_{2} = \frac{1}{{\left( {2\omega_{o} } \right)^{2} \cdot L_{2} }}$$

where ${\omega }_{o}$ is the angular frequency.

**Figure 3:**
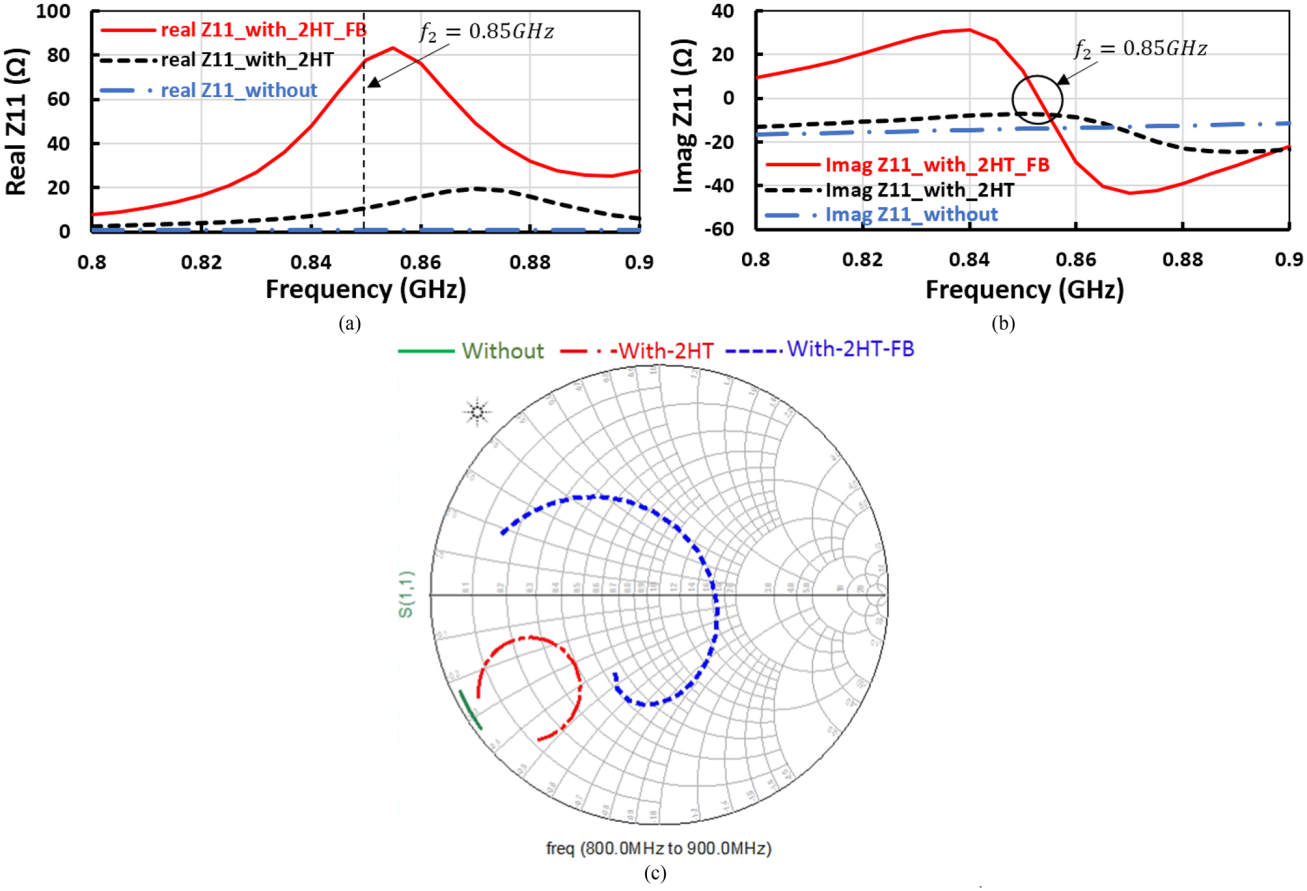
Input impedance, (**a**) Real part, (**b**) Imaginary part, and (**c**) Smith chart in three cases without matching; with 2nd harmonic termination (2HT), and with 2HT in addition to feedback network (FB).

Figure 3 illustrates the effect of the second harmonic resonance and feedback circuits on the proposed positive voltage doubler, where Fig. 3a,b demonstrate real and imaginary parts of input impedance, while Fig. 3c displays the smith chart of input impedance, without any thing the proposed design is un-matching, when insert the second harmonic termination (2HT) circuit that added inductive reactance to the circuit and improve the input matching as illustrated in Fig. 3 and when insert the feedback (FB) network that enhance the matching as displayed in Fig. 3.

${C}_{b}$ is DC blocking capacitor and its value is very big in microfarad for not to influence the input matching. The input matching network is implemented to match 50 Ω with the voltage doubler diode’s input impedance. It includes radial stubs and a micro-strip inductor, where the radial stub works as a capacitor and enhances the rectifier performance by improving the DC output voltage, increasing conversion efficiency, and enhancing the input return loss. The micro-strip inductor potted in Fig. 1b, in which the inductance and the quality factor are calculated by Eqs. (3) and (4) in^21,22^,

3 $$L_{inductor} = \left( {Im\left( {4/\left( {Y_{11} + Y_{22} - Y_{12} - Y_{21} } \right)} \right)} \right)/\omega$$

4 $$Q_{inductor} = - \frac{{Im\left( {Y_{11} + Y_{22} - Y_{12} - Y_{21} } \right)}}{{Re\left( {Y_{11} + Y_{22} - Y_{12} - Y_{21} } \right)}}$$

where ${Y}_{11}$ is the input driving point admittance, ${Y}_{12}$ is the forward transfer admittance, ${Y}_{21}$ is the reverse transfer admittance, and ${Y}_{22}$ is the output driving point admittance.

**Figure 4:**
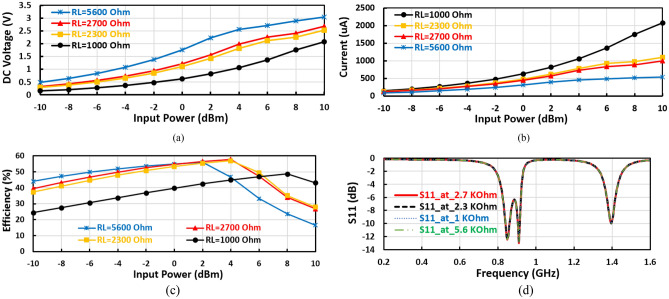
(**a**) DC output voltage, (**b**) DC output current, (**c**) conversion efficiency, and (**d**) Input reflection coefficient ${(S}_{11})$ at several load resistance (${R}_{L}$) of the positive voltage doubler.

The micro-strip inductor has an inductance of 12 nH and a quality factor of about 65 and is used to adjust the matching circuit at 1400 MHz. The micro-strip inductor has a length of 21.2 $$\mathrm{mm}$$ employing variable width from 0.25 to 0.4 $$\mathrm{mm}$$ to improve the inductor quality factor, where the radial stubs and micro-strip inductor are used to match the diodes input impedance to 50 Ω at 1400 MHz frequency band. The series resonance feedback and parallel resonance to the ground are used to generate an extra frequency band at 850 MHz. The value of the load resistance $${(R}_{L})$$ was chosen to achieve the required output DC voltage ($${V}_{DC}$$), and the desired output current with acceptable (Maximum) RF–DC efficiency (η). The output DC voltage ($${V}_{DC}$$) rises with $${R}_{L}$$ until the DC output voltage becomes constant at a high RF input power. The behavior of the conversion efficiency in terms of load resistance is very complicated where it increases with $${R}_{L}$$ reaching a maximum and decreases again as *R*_*L*_ increases. Figure 4a–c display the output DC voltage, the DC output current, and the RF–DC efficiency against the input power at several values of load resistance ($${R}_{L}$$) of the suggested voltage doubler. The first proposed RF voltage doubler achieves an excellent performance over a wide range of terminal resistance $${R}_{L}$$ from 1000 to 5600 Ω with a slight adjustment, as illustrated in Fig. 4. The desired DC output voltage and the required DC output current are obtained at load resistance of 2700 Ω at 0 dBm RF input power. The load terminal resistance has no effect or slight effect unnoticeable on the input reflection coefficient $${(S}_{11})$$, Fig. 4d illustrates the input reflection coefficient $${(S}_{11})$$ at several values of load resistance as displayed in the figure, the input return loss ($${S}_{11})$$ is not influence by change the value load terminal resistance. But changing the load resistance has the main effect on the output current from the proposed voltage doubler, based on the required output current the load resistance is selected.

### Fabrication and experimental results of first voltage doubler

**Figure 5:**
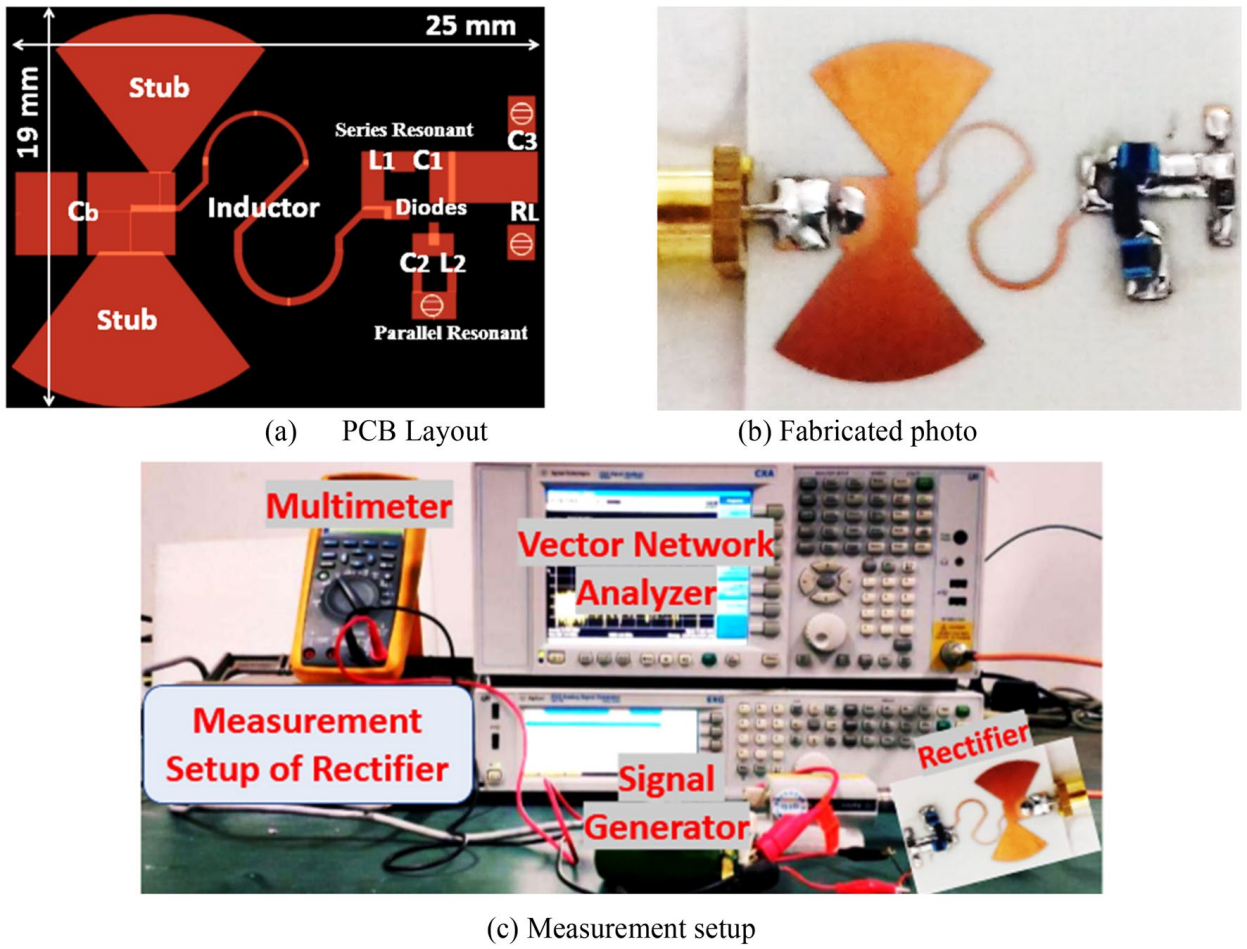
Proposed Fabricated RF voltage doubler and measurement setup.

**Figure 6:**
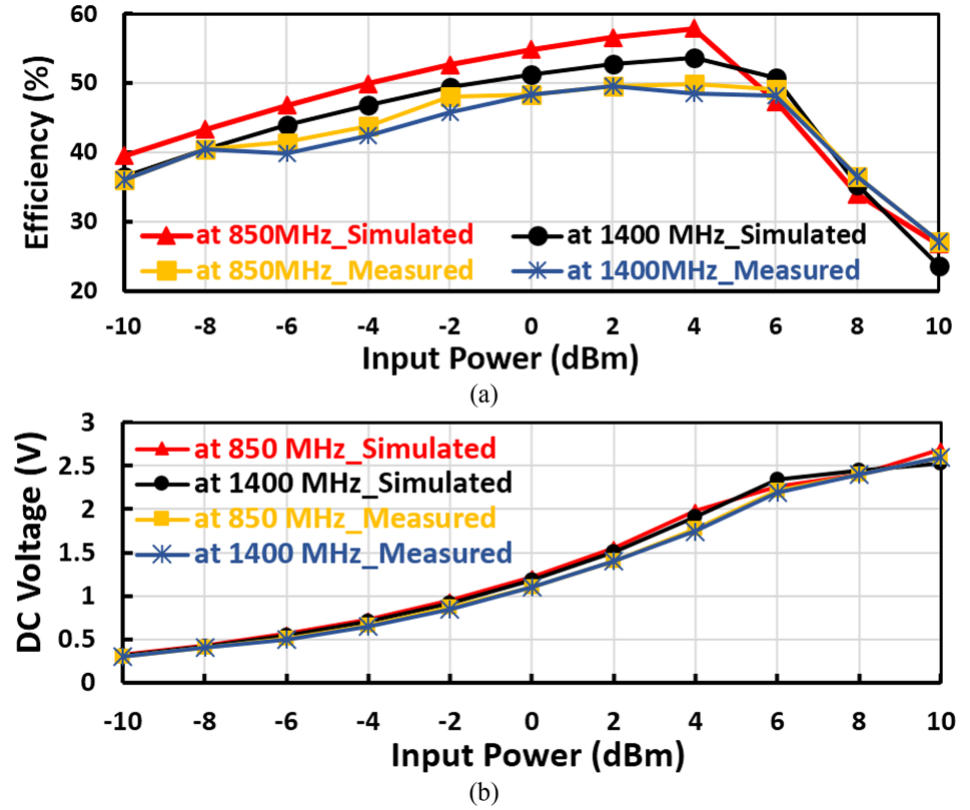
(**a**) Measured Conversion efficiency and (**b**) DC output voltage with radio frequency input power of the first suggested voltage doubler.

The suggested RF voltage doubler is implemented utilizing Roger material (RO4003C) of Epson 3.38 and thickness 0.81 $\mathrm{mm}$ and simulated by ADS for circuit simulation, and ADS Momentum simulation for circuit layout and physical verifications. The layout of the suggested RF voltage doubler is illustrated in Fig. 5a, where the size of the RF voltage doubler is 4.7 ${\mathrm{cm}}^{2}$ while Fig. 5b,c are the fabricated prototype and measurement setup of the proposed voltage doubler. The reflection coefficient (${S}_{11}$) of the fabricated rectifier was observed using vector network analyzer (VNA) with part number (ZVA67). While the DC output voltage was measured by a digital multimeter where the RF input power was supplied from the signal generator of model number (MG3710A) as shown Fig. 5c. The conversion efficiency versus the input power of the suggested RF voltage doubler is displayed in Fig. 6a, where the peak simulated efficiency equals 59% at 4dBm input power and around 54% at 0 dBm input power. The DC output voltage against RF input power is shown in Fig. 6b, where the constant DC output voltage is about 2.5 V at 6 dBm input power, whereas the DC output voltage at 0 dBm input equals about 1.2 V.

The conversion efficiency is the ratio between the output power and the input power, where the RF–DC conversion efficiency is calculated utilizing the following equation.

5 $$\eta = \frac{{P_{out} }}{{P_{in} }} = \frac{{{ }V_{DC}^{2} }}{{P_{in} {*}R_{L} }}$$

where $${V}_{DC}$$ is DC output voltage, $${R}_{L}$$ is load terminal resistance, $${P}_{in}$$ is RF input power, and $${P}_{out}$$ is output power that equals the ratio between square DC output voltage and load resistance ($${{V}_{DC}}^{2}/{R}_{L})$$. As demonstrated in Eq. (5), the conversion efficiency is directly proportional to the output DC voltage ($${V}_{DC}$$) and inversely proportional to the RF input power ($${P}_{in}$$), when the output DC voltage has become saturated, the conversion efficiency will be reduced with an increase in the input power as displayed in Fig. 6. There is a reduction in the RF–DC efficiency after 4 dBm because the output DC voltage of the suggested voltage doubler becomes constant after RF input power of 6 dBm as shown in Fig. 6b. When the output DC voltage saturates while increasing the RF input power, the conversion efficiency will reduce as plotted in Fig. 6a and this can be known concerning Eq. (5).

**Figure 7:**
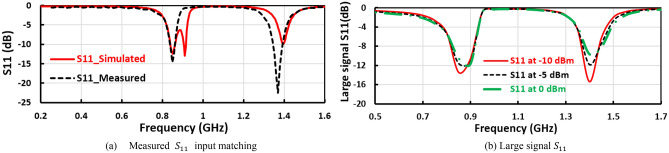
Measured input reflection and large signal ${S}_{11}$ of the first voltage doubler.

**Figure 8:**
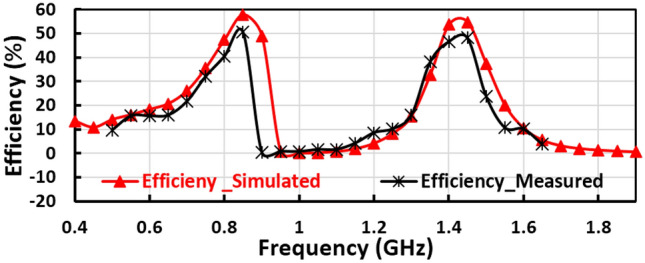
Measured Conversion efficiency of the first proposed voltage doubler.

The measured and simulated input reflection coefficient (${S}_{11}$) of the suggested voltage doubler is displayed in Fig. 7a, where the voltage doubler operates in two frequency bands at 850 MHz and 1400 MHz. The large signal input reflection coefficient ${(S}_{11})$ with the frequency is plotted in Fig. 7b, at 0 dBm, − 5 dBm, and − 10 dBm RF input power levels. The measured and simulated RF to DC conversion efficiency with the frequency of the voltage doubler is displayed in Fig. 8, at radio frequency input power level equals 4dBm the peak simulated conversion efficiency equal 59% and 56% for 850 MHz and 1400 MHz frequency bands, respectively as illustrated in Fig. 8. The discrete components selection, especially the inductor, is based on the high-quality factor at the desired frequency. To ensure excellent matching between the measured and simulated results, the measured scattering parameters file (SNP file) of the selected lumped components are inserted in the simulation program.

**Figure 9:**
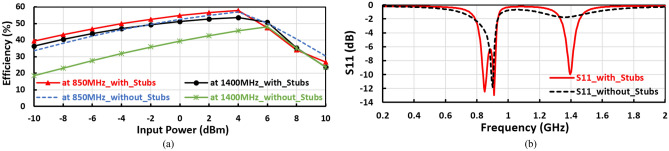
(**a**) Conversion efficiency versus input power for the suggested positive voltage doubler with stubs and without stub, and (**b**) effect of the stubs on the input reflection coefficient (S_11_) for the suggested positive voltage doubler.

The radial stubs and the designed inductor used to match the first band at ${f}_{1}$, where the radial stub effects as a capacitor to enhance the design achievement by rising the DC voltage, conversion efficiency, and enhancing the input reflection coefficient ${(\mathrm{S}}_{11})$. The stubs and the designed inductor are specially used to match the proposed voltage doubler at frequency band of ${f}_{1}=1400 \mathrm{MHz}$, wherefore if the stubs do not exist these have poor influence on the performance of 1400 MHz frequency band. The influence of the stubs on the suggested positive voltage rectifier is plotted in Fig. 9a,b. The stubs improve the conversion DC–RF efficiency of the suggested design by more than 15% and enhance the input reflection coefficient $({S}_{11})$ to be matched at frequency band of 1400 MHz.

Where the stub effects as a capacitor, enhances the achievement of the design such as improving the RF–DC conversion efficiency and the input reflection coefficient $({S}_{11})$ as plotted in Fig. 9a,b as well as raising the DC voltage at the expanse of the design size. When the stub size increases, the voltage doubler achievement improves, and the stub size parameters are characterized by length, bottom width, and edge angle.

## Proposed negative DC supply voltage

### Proposed negative voltage rectifier design

**Figure 10:**
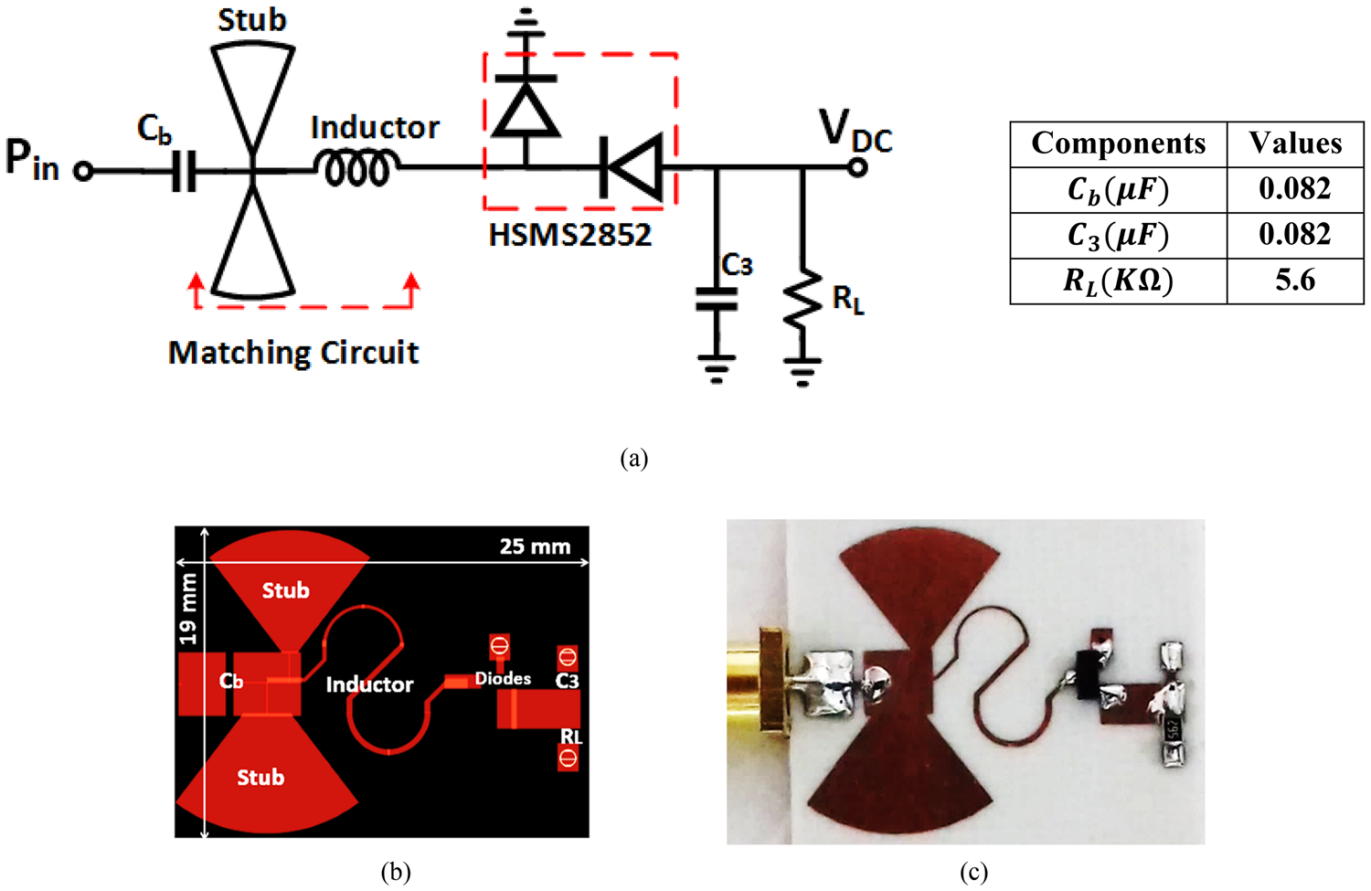
(**a**) Schematic circuit of the proposed negative voltage rectifier, (**b**) layout, and (**c**) fabricated Prototype photography.

**Figure 11:**
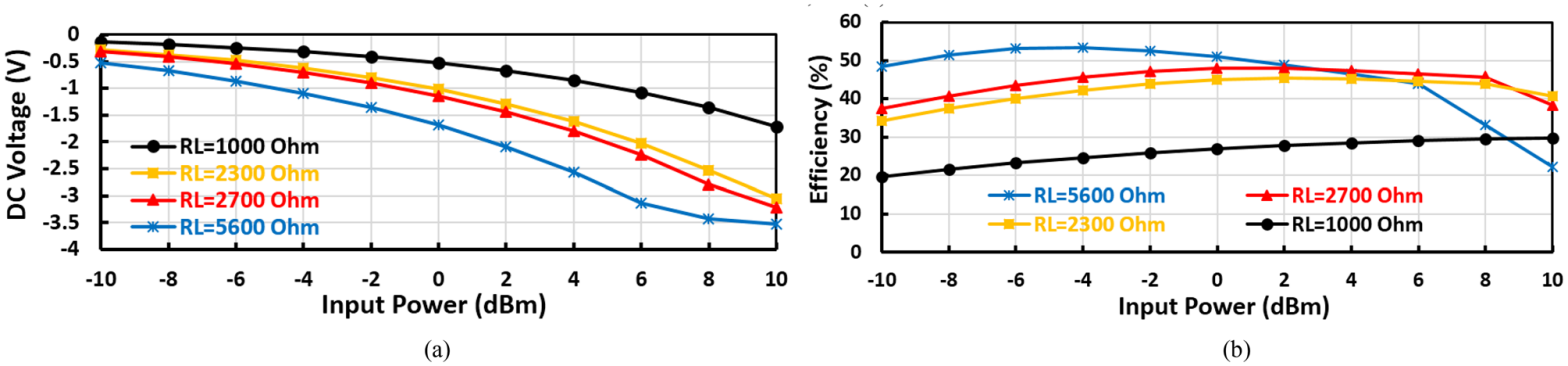
(**a**) DC output voltage and (**b**) RF-DC conversion efficiency at several load resistance (R_L_) of the suggested negative voltage rectifier.

The proposed negative voltage rectifier consists of input matching (radial stubs and a micro-strip inductor), and voltage doubler diodes where the output is connected to the negative diode terminal (anode terminal) as displayed in Figs. 10a and 11a,b show the DC output voltage and the RF–DC conversion efficiency against the input power at several load terminal resistance (${R}_{L}$). According to our criteria of obtaining the highest efficiency and the highest possible output voltage at the low input power range, the negative voltage rectifier then has its optimum load terminal resistance equals 5600 Ω. The selection of load resistance to achieve the required output DC voltage with extreme efficiency, with no condition on the output current so that the proposed negative voltage doubler has better efficiency.

### Fabrication and experimental results of negative voltage rectifier

**Figure 12:**
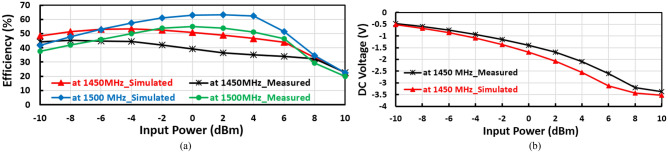
(**a**) RF–DC conversion efficiency and (**b**) Negative output DC voltage against input power of the suggested negative voltage rectifier.

**Figure 13:**
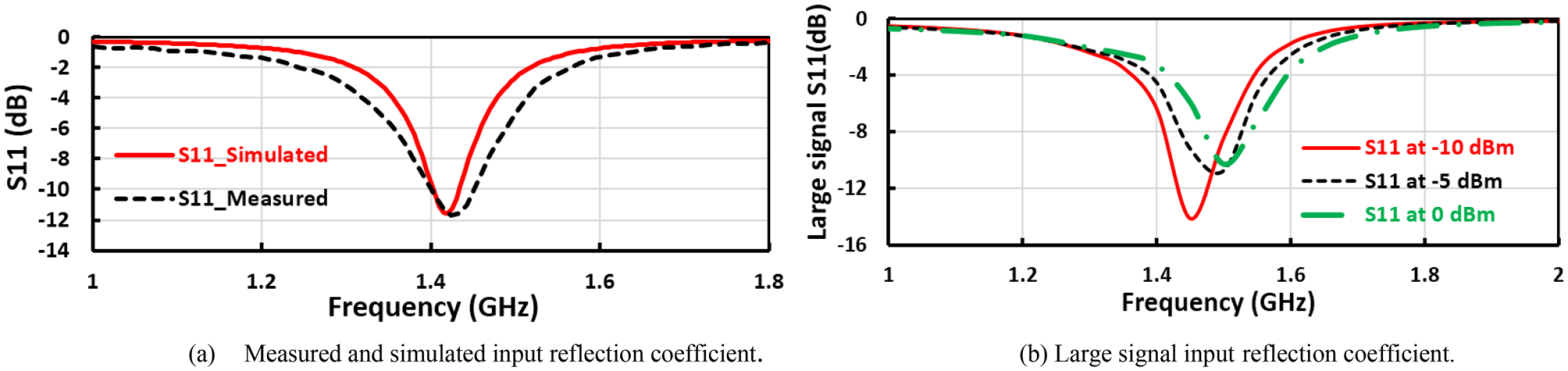
Input return loss at 1450MHz of the negative voltage rectifier.

**Figure 14:**
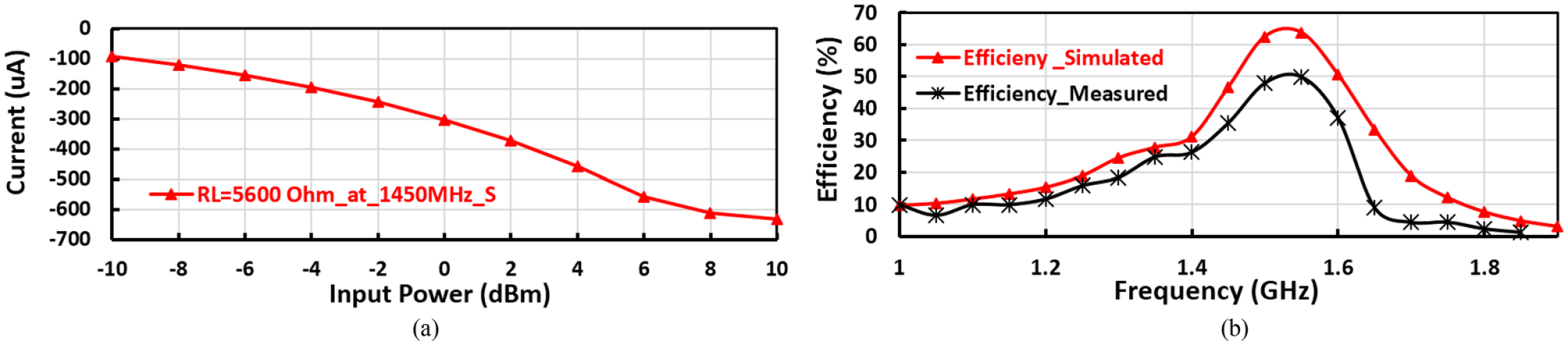
(**a**) Negative Output DC current against input power and (**b**) Measured RF–DC conversion efficiency with frequency of the suggested negative voltage rectifier.

The layout of the suggested negative voltage rectifier is illustrated in Fig. 10b, including a blocking capacitor, input matching circuit, and voltage doubler. The size of the negative voltage rectifier is 4.7 cm^2^. The manufactured prototype photography of the proposed negative voltage rectifier is plotted in Fig. 10c. The RF–DC conversion efficiency against the input power of the suggested negative voltage rectifier is displayed in Fig. 12a, as illustrated, the peak conversion efficiency equals 65% at 2 dBm RF input power and around 50% at − 10 dBm RF input power. The negative output DC voltage versus RF input power is illustrated in Fig. 12(b), where the constant negative output DC voltage of − 3.5 V, − 1.2 V, and − 0.5 V at 6 dBm, − 2 dBm, and − 10 dBm input power, respectively. The input matching ${(S}_{11})$ of the suggested negative voltage rectifier is plotted in Fig. 13a, where the suggested negative voltage rectifier operates the frequency band at 1450 MHz. The large-signal input return loss ${(S}_{11})$ at the 1450 MHz frequency band of the negative voltage rectifier is displayed in Fig. 13b at high input power levels equal to 0 dBm, − 5 dBm, and − 10 dBm. The current against the input power of the proposed negative voltage rectifier is displayed in Fig. 14a. The measured and simulated results of the RF–DC conversion efficiency with the frequency of the suggested negative voltage rectifier plotted in Fig. 14b, at RF input power equals 4 dBm, the maximum measured efficiency equals 50%.

**Figure 15:**
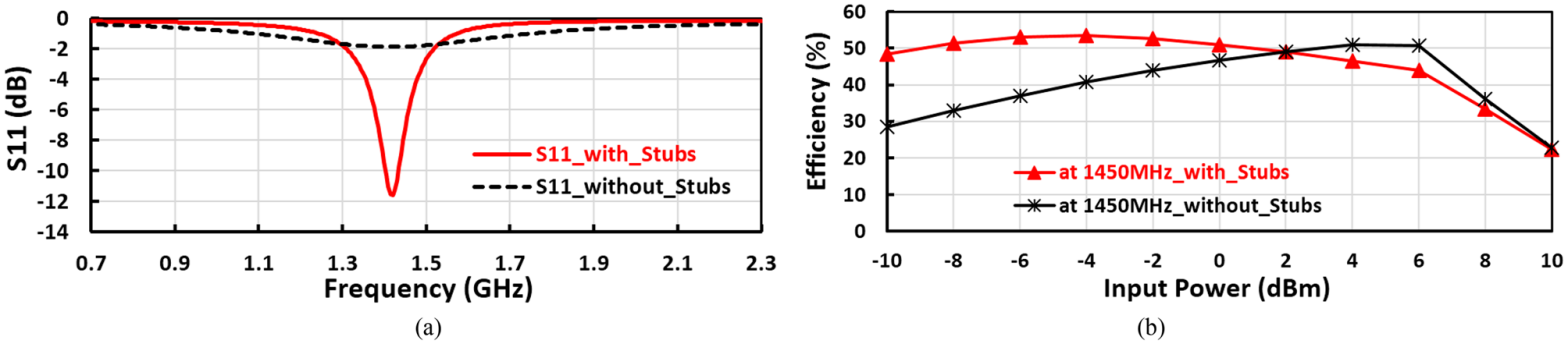
(**a**) Effect of the stubs on the input reflection coefficient ${(S}_{11})$ for the suggested negative voltage doubler, and (**b**) Conversion efficiency versus input power for the suggested negative voltage doubler with stubs and without stubs.

The input matching of the negative voltage doubler design consists of the stubs and the designed inductor, so the absence of the stubs has a large influence on the input reflection coefficient ($${S}_{11})$$ and also affected on the efficiency, as displayed in Fig. 15a The design is unmatched without the stubs, consequently, the efficiency is decreased by about 20% in the case when the stubs do not exist as illustrated in Fig. 15b.

**Figure 16:**
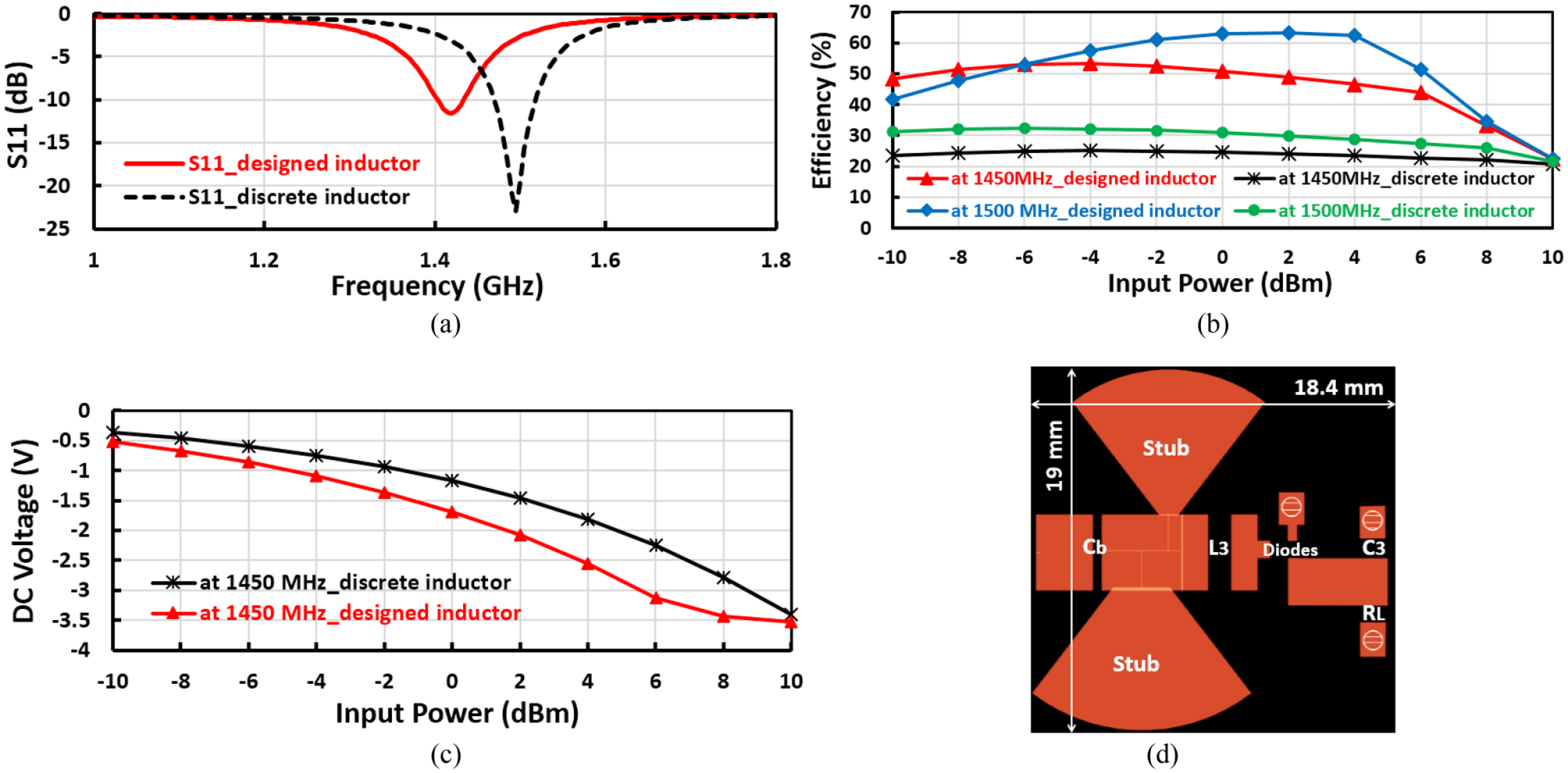
Effect of using the discrete lumped inductor in the negative voltage doubler; (**a**) Input reflection coefficient ${S}_{11}$, (**b**) Efficiency, (**c**) DC output voltage, and (**d**) RF layout.

**Figure 17:**
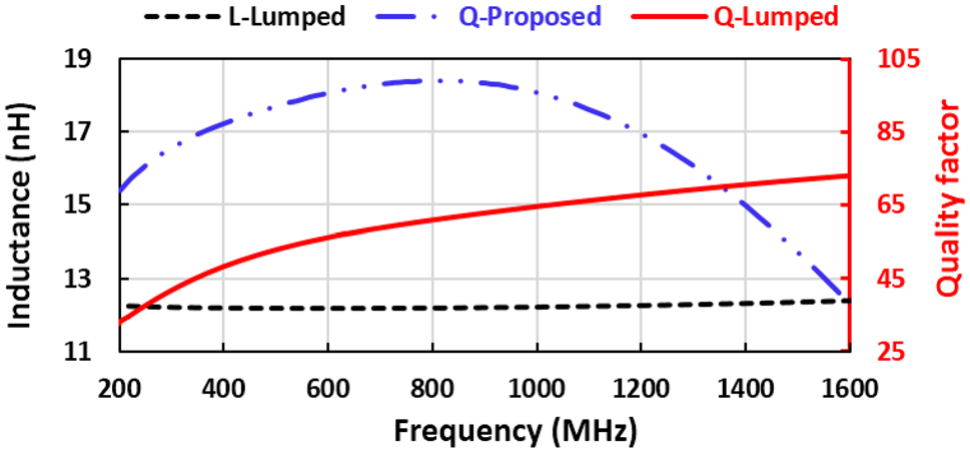
Discrete lumped inductor inductance, quality factor, and the proposed inductor quality factor.

The input matching in the proposed designs especially at the frequency band of 1450 MHz consists of the stubs and the designed inductor. This way is simple and does not need discrete components, the designed inductor has high quality over a wide frequency range, and the input matching circuit is more accurate and occupied less area than the PI or T-matching method, the designed elements are more accurate than the discrete lumped component because the component tolerance. The proposed novel negative voltage doubler is used as a negative bias voltage in mm-wave CMOS receiver and it is designed to obtain high efficiency and large DC output voltage at ambient power so that designed high-quality factor and large SRF inductor and also design the stubs to enhance RF performance. Furthermore, the designed input matched is more accurate than using the discrete lumped components because no tolerance in the components value, no manufacturing error, high Q-factor, and large SRF, all these reasons tend to propose negative voltage doubler design has more benefits. Figure 16 illustrates the effect of using the discrete lumped inductor in the proposed negative voltage doubler, where the input reflection coefficient ${(S}_{11})$ enhanced and shifted-up by using discrete inductor as shown in Fig. 16a, whereas the efficiency and output DC voltage decreased by large values as displayed in Fig. 16b,c and the PCB size reduced as explained in Fig. 16d for layout. Figure 17 displays the discrete lumped inductor inductance, quality factor, and the proposed designed inductor quality factor, where the discrete lumped inductance and quality factor are gotten from S2P file provided by the manufacturing company. From Fig. 17 the proposed designed inductor achieves high quality factor than the discrete lumped inductor. The comparison between using the designed inductor and discrete lumped inductor is illustrated in Table 1.

**Table 1:** Comparative study between the negative voltage doubler using designed inductor and discrete inductor.

Index	Negative VD with designed inductor	Negative VD with discrete inductor
Efficiency@-10 dBm (%)	48.5	33
${V}_{DC}$@-10 dBm (%)	-0.52	− 0.36
${S}_{11}$(dB)	-7.2	− 5.9
PCB size ${\mathrm{cm}}^{2}$	4.7	3.5
Inductor quality factor	> 65	> 35
Inductor SRF (GHz)	1.85	6
Inductor part number	Designed inductor	LQW18AN13NG00

The proposed designs exhibit an acceptable agreement between the simulated and measured results. The slight error comes from components tolerance, SMA soldering, components soldering, and Fabrication tolerance. The suggested radio frequency voltage doubler and negative voltage rectifier display comparable or even superior achievement to the reported radio frequency voltage doubler and radio frequency rectifier fabricated using micro-strip materials in Table 2. As illustrated in Table 2, the proposed fabricated rectifiers achieve the highest efficiency and DC output voltage at the lowest input power of − 10 dBm and 0 dBm compared to the recently published works so that they can be used in low-power applications as the energy harvesting applications. Furthermore, the suggested fabricated designs exhibit the best input matching of less than -15dB and the output current is taken into consideration. Finally, the proposed manufactured rectifiers are interested by the output current external from the rectifiers, where the suggested designs are used as real DC voltage supplies for mm-Wave CMOS receivers. Where the output current of the proposed positive and negative voltage doubler rectifiers are 450 uA and 100 uA respectively.

**Table 2:** Performance comparison of voltage doubler and negative voltage rectifier integrated using micro-strip materials.

Index	This work		^4^		^5^	^6^	^9^	^13^	^14^	^15^	^16^	^17^
Topology	Voltage doubler	Negative voltage	Dual-band		Broadband		Wideband	Dual-band	Single-band	Single-band	Single-band	Dual-band
Freq. (GHz)	0.85 and 1.4	1.45	0.65 and 0.9		1–2.4	0.87–2	0.7–1.1	0.49 and 0.86	0.868	0.673	0.915	1.88 and 0.9
Substrate	RO4003C		RO4003C		NA	FR-4	RO4003C	FR-4	NA	FR-4	FR-4	FR-4
Eff (%) @ dBm	50@0	45@-10	60.5@2	45@4	50@4	40@0	69@3	17.3%@-10 7.5%@-10	22.5 @-19	40@-20	44@-10	45@-7 33@-7
Load (${R}_{L}$)KΩ	2.7	5.6	10	22	1.6	2	10	11	2.193	1	3.6	1.5
Output DC voltage @dBm	1.2V@0	− 0.5V@-10	1@-10	0.7@-10	1V@0	NA	3.4@3	0.43@-10 0.28@-10	NA	0.245@-20	0.136	0.3@-7
Input return loss	< − 15 dB	< − 12 dB	< − 12 dB		− 10 dB	− 5 dB	< − 10 dB	< − 10 dB	< − 10 dB	< − 30 dB	< − 10 dB	NA
PCB area (${\mathrm{cm}}^{2}$)	4.7	4.7	2.8	3.6	10	11.4	1.26	NA	NA	NA	NA	10.4

## Conclusion

Two energy harvesting circuits that generate positive and negative supply voltage are implemented and they are appropriate to the bias voltage of the mm-wave receiver. The first introduced harvesting circuit is a positive voltage double with parallel resonance and series resonance feedback networks and works in dual frequency bands of 850 and 1400 MHz. While the second suggested harvesting network is a negative voltage rectifier with input matching radial stubs, micro-strip inductor, and Schottky diode in opposite polarity to produce the negative voltage required in the mm-wave receiver.

## Data Availability

The datasets used and/or analysed during the current study available from the corresponding author [Marwa Mansour] on reasonable request.
